# Preparation of Nanocomposites for Antibacterial Orthodontic Invisible Appliance Based on Piezoelectric Catalysis

**DOI:** 10.3390/s23115336

**Published:** 2023-06-05

**Authors:** Yingying Shi, Ningning Zhang, Jiajie Liu, Junbin Wang, Shuhui Shen, Jingxiang Zhang, Xiaoli An, Qingzong Si

**Affiliations:** 1 School of Stomatology, Lanzhou University, Lanzhou 730030, China; shiyy20@lzu.edu.cn (Y.S.); zhangnn21@lzu.edu.cn (N.Z.); jjliu21@lzu.edu.cn (J.L.); wangjb2016@lzu.edu.cn (J.W.); 220220929390@lzu.edu.cn (S.S.); anxl@lzu.edu.cn (X.A.); 2 School of Civil Engineering and Mechanics, Lanzhou University, Lanzhou 730030, China; zhangjx19@lzu.edu.cn

**Keywords:** BaTiO_3_NPs, antibacterial, orthodontic invisible appliance, piezoelectric catalysis

## Abstract

Compared to fixed orthodontic appliances with brackets, thermoplastic invisible orthodontic aligners offer several advantages, such as high aesthetic performance, good comfort, and convenient oral health maintenance, and are widely used in orthodontic fields. However, prolonged use of thermoplastic invisible aligners may lead to demineralization and even caries in most patients’ teeth, as they enclose the tooth surface for an extended period. To address this issue, we have created PETG composites that contain piezoelectric barium titanate nanoparticles (BaTiO_3_NPs) to obtain antibacterial properties. First, we prepared piezoelectric composites by incorporating varying amounts of BaTiO_3_NPs into PETG matrix material. The composites were then characterized using techniques such as SEM, XRD, and Raman spectroscopy, which confirmed the successful synthesis of the composites. We cultivated biofilms of *Streptococcus mutans* (*S. mutans*) on the surface of the nanocomposites under both polarized and unpolarized conditions. We then activated piezoelectric charges by subjecting the nanocomposites to 10 Hz cyclic mechanical vibration. The interactions between the biofilms and materials were evaluated by measuring the biofilm biomass. The addition of piezoelectric nanoparticles had a noticeable antibacterial effect on both the unpolarized and polarized conditions. Under polarized conditions, nanocomposites demonstrated a greater antibacterial effect than under unpolarized conditions. Additionally, as the concentration of BaTiO_3_NPs increased, the antibacterial rate also increased, with the surface antibacterial rate reaching 67.39% (30 wt% BaTiO_3_NPs). These findings have the potential for application in wearable, invisible appliances to improve clinical services and reduce the need for cleaning methods.

## 1. Introduction

Malocclusion is a developmental disorder of the craniofacial complex that affects the jaws, tongue, and facial muscles and is known as one of the three major oral diseases that affect human oral function, aesthetics, social interactions, and health-related quality of life [1]. Malocclusion represents a high prevalence, ranging from 20% to 100%, as reported by different researchers [2,3,4,5]. Several studies have indicated that fixed orthodontic treatment can have adverse effects on the oral health of patients with malocclusion. These effects can range from mild to severe and include issues such as enamel demineralization, white lesions, caries, periodontics, and halitosis [6,7,8]. Consequently, traditionally fixed orthodontic treatment is no longer adequate for meeting the orthodontic needs of modern patients. Aesthetic considerations play a crucial role in the practice of dentistry. In addition, studies have shown that thermoplastic invisible orthodontic aligners pose fewer risks to oral hygiene compared to traditionally fixed orthodontic treatments [6], which has resulted in the thermoplastic invisible orthodontic appliances being widely used in the field of orthodontics. Thermoplastic invisible orthodontic aligners are produced through a process that involves heating thermoplastic sheets and molding them onto dental cast models using either vacuum or pressure forming. Several thermoplastics, such as polyethylene, polycarbonate, polypropylene, and polyethylene terephthalate glycol-modified (PETG), are presently utilized for the preparation of these appliances [9,10,11]. Notwithstanding their benefits, thermoplastic, invisible orthodontic appliances tend to promote bacterial colonization. Sibel Tektas et al. found that cariogenic bacteria such as *S*. *mutans* and Lactobacillus were more likely to accumulate on the surface of macromolecular, invisible appliances when observed under an electron microscope [12]. Moreover, extensive literature suggests that during the course of invisible orthodontic treatment, these appliances remain in contact with the tooth surface for prolonged periods, impairing the self-cleaning mechanism of the teeth and increasing the risk of bacteria-related conditions, including enamel demineralization, dental caries, and periodontal disease [9,13,14].

To address the issues mentioned above, several interventions have been proposed. Levrini Luca et al. [15] suggest that brushing associated with the use of effervescent tablets containing sodium carbonate and sulfate is the most effective method of cleaning thermoplastic aligners. Nonetheless, this approach fails to comprehensively eliminate biofilm from the orthodontic aligner surfaces. Biofilm may persist to some extent, especially on the internal surfaces, potentially leading to discoloration, unpleasant odor, and synergistic interactions with bacteria already present in the oral cavity. In addition, Park Sohyeon et al. [9] developed an antibacterial multilayer film based on polysaccharides for the surface of the invisible orthodontic appliances, and found that the bacterial inhibition rate increased by 75%, but its mechanical properties decreased, and the multilayer film had a collapsed porous structure and cracks after the thermal forming process. Therefore, additional measures are necessary to overcome these limitations of thermoplastic, invisible orthodontic aligners.

The piezoelectric effect was discovered in 1880 by the brothers Pierre Curie and Jacques Curie. Piezoelectric materials exhibit a distinctive property whereby they generate an electric field and surface piezopotential upon being subjected to an external mechanical stress [16]. They have been widely used in implanted biosensors, wearable devices, and semiconductor piezocatalysis [17,18,19]. Barium titanate (BaTiO_3_) is a chemically simple and environmentally benign material [20]. Given their attractive piezoelectric properties, BaTiO_3_-based systems have also been found to be useful in biological applications, including intravascular ultrasonic transducers for biomedical imaging applications [21], biocompatible nanogenerators for application as in vivo power sources [22], biocompatible composites for use in bone regeneration [23], and scaffolds to support cellular survival and proliferation for potential use in personalized bone implants [24]. There have also been attempts to utilize BTO-based systems for antibacterial and oral applications. For example, previous studies with BaTiO_3_NPs have shown moderate antibacterial properties and antibiofilm properties [25,26,27]. It is important to note that piezoelectric materials are ultrasensitive to mechanical vibration, even water flow, muscle movement, and respiration can also induce electrical charges [28,29,30]. Moreover, Carolina Montoya et al. [31] have shown that bacterial inactivation on charged surfaces may be due to electrolysis of molecules on the surface of bacterial cells, resulting in the production of toxic substances, such as hydrogen peroxide, chlorine molecules, and oxidative radicals. These surface charges also contribute to the production of ROS in cells, such as superoxide anion (O^2−^), hydrogen peroxide (H_2_O_2_), hydroxyl radical (•OH), lipid hydroperoxides, and singlet oxygen (O_2_^1^). The increase in reactive oxygen species exceeds the antioxidant capacity of the cell, which will produce oxidative stress in the bacteria, damage lipids, proteins, and DNA, and eventually lead to cell death. Yang Wang et al. [32] have shown that due to the compressive stress based on the piezoelectric effect, additional charges are released from the surface, dispersed into the solution, become free charges, and combine with water molecules to produce reactive species such as hydroxyl radicals (•OH) and superoxide anions (O^2−^). Atul Dhall et al. have shown that the negatively charged BaTiO_3_NPs may prevent the adhesion of negatively charged *S. mutans*. Additionally, previous studies have shown that BaTiO_3_ nanocomposites possess favorable biocompatibility, making them a suitable platform for biomaterials [27].

In this study, we have developed, for the first time, a dental composite device with self-powering and antibacterial properties, designed to be worn as an orthodontic aligner and retainer. These appliances can exhibit catalytic antibacterial effects by responding to minor mechanical stimulations caused by routine oral activities (e.g., speaking, drinking, and exercising) without the need for any auxiliary devices. The continuous production of active radicals by the appliances will effectively target bacteria and prevent biofilm formation on tooth surfaces. Furthermore, as the appliances do not require an external energy source, such as photostimulation or ultrasonic agitation, their antibacterial activity is a spontaneous process. Additionally, minor mechanical stimulations in the oral environment are ongoing, ensuring a long lifespan for the appliances.

## 2. Materials and Methods

### 2.1. Fabrication of Nanocomposites Diaphragm

**Figure 1:**
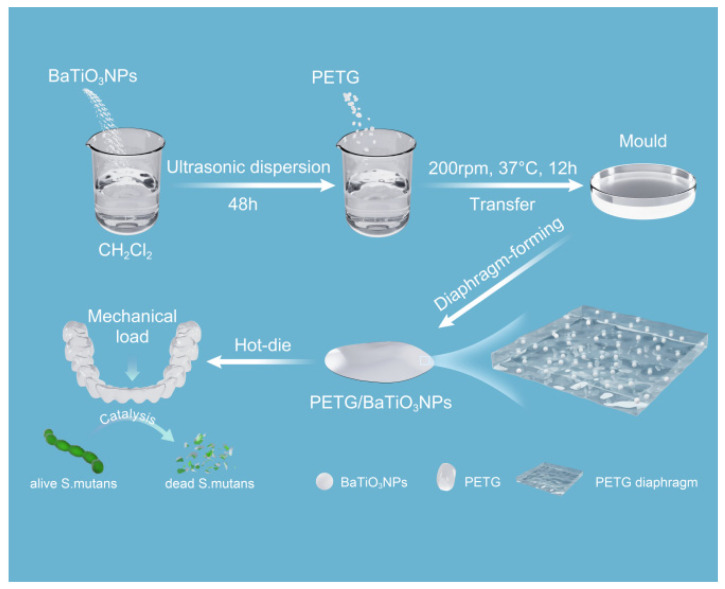
Schematic diagram of synthesis of PETG/BaTiO_3_NPs nanocomposites diaphragm.

As schematically illustrated in Figure 1, the nanocomposites diaphragm was synthesized through a solution blending method. Initially, BaTiO_3_NPs (Shanghai Lin En Chemical Technology Co., Ltd., Shanghai, China) with varying mass fractions (10 wt%, 20 wt%, and 30 wt%) were introduced into dichloromethane, an organic solvent. The resulting mixture was subjected to ultrasonic dispersion for 48 h. PETG matrix particles ( SK JN200, Seoul, Republic of Korea) with different mass fractions were then added to the solution system, followed by sealing the container. The mixed slurry was transferred to a mold for performing diaphragms after shaking in a constant temperature shaker at 200 rpm and 37 °C until completely dissolved, and 10 wt%, 20 wt%, and 30 wt% PETG/BaTiO_3_NPs composite diaphragms were prepared. The PETG diaphragm was also synthesized to serve as a control group: a certain amount of PETG matrix particles were dissolved in dichloromethane organic solvent and sealed the container at 200 rpm, 37 °C constant temperature oscillation, until completely dissolved. The slurry was transferred to a mold for performing diaphragm. Finally, some samples were cut into circular sheets with a diameter of 0.5 cm, covered with conductive adhesive, and then subjected to polarization treatment in a high-pressure polarization device at 10 KV/mm for 20 min.

### 2.2. Characterization

The nanocomposites diaphragm microstructure was characterized by scanning electron microscopy (SEM; Hitachi S-4800, Tokyo, Japan), X-ray diffraction (XRD; Rigaku D/MAX-2400, Tokyo, Japan), and Raman spectra (HORIBA, XPloRAPLUS, Kyoto, Japan). First, the BaTiO_3_ nanoparticles and fracture surface of PETG/BaTiO_3_ (30 wt%) nanocomposites were analyzed and characterized using a scanning electron microscope. Images were captured at magnifications ranging from 100 to 50,000 and an acceleration voltage of 10 KV. To enhance the conductivity, all samples were coated with a thin layer (20–30°) of gold via sputtering. Then, the presence of phases in both PETG diaphragm and PETG/BaTiO_3_NPs (30 wt%) nanocomposite diaphragm was investigated using X-ray diffraction (XRD) technique. The diffraction angle range of 10–80° was utilized for this purpose. The Raman spectra of both PETG diaphragms and PETG/BaTiO_3_NPs (30 wt%) nanocomposites diaphragms were obtained using a Raman microscope equipped with an Olympus microscope and a 532 nm laser. The Raman spectra were obtained by scanning 32 times in two seconds.

### 2.3. Contact Angle and Salivary Absorption

The contact angle of PETG diaphragm and PETG/BaTiO_3_NPs composite diaphragm was determined using a contact angle measurement setup with 1 mL of deionized water. The sample is polished with ultra-fine sandpaper prior to testing to achieve a consistent surface roughness. For measuring the salivary absorption rate, test samples were prepared by making PETG diaphragms and PETG/BaTiO_3_NPs composite diaphragms, drying them, and recording their weight. The samples were then placed into artificial saliva at 37 °C and left to soak for different time periods of 1 h, 3 h, 6 h, 12 h, 24 h, 48 h, 72 h, and 168 h. After soaking, the samples were quickly dried and weighed separately.

### 2.4. Mechanical Properties

Microhardness tester was used to determine the microhardness of samples. Four groups of samples to be tested were PETG diaphragm and PETG/BaTiO_3_NPs composite diaphragm, and 6 parallel samples were prepared in each group. The microhardness tester was loaded at 200 N for 10 s. After the diamond press head was pressed into the sample, a pit was created on the surface of the sample. The diagonal length of the pit was measured by the eyepiece micrometer. The microhardness values of the samples were calculated according to the load and the diagonal length of the pits. Five points were tested for each sample, and the average value was obtained at the end. In addition, according to the thickness of the test sample GB6672, the thickness of each sample is measured at three points within the range of marking, and the average value is taken. After marking the sample, the sample is clamped, and the longitudinal axis of the sample is required to coincide with the center line of the upper and lower fixtures of the tester, and the tightness is appropriate. The tensile test is conducted at a speed of 5 mm/min.

### 2.5. In Vitro Antibacterial Test

We established an in vitro model using a single-species biofilm to assess the antibacterial effectiveness of PETG/BaTiO_3_NPs composites. We selected *Streptococcus mutans* as our bacterial strain due to their strong correlation with dental caries and enamel demineralization. The bacterial strain was cultivated on a brain-heart infusion (BHI) agar plate and incubated at 37 °C for 24 h. A single colony was then collected and incubated in fresh BHI while being stirred at 200 rpm overnight. The resulting solution was diluted in fresh BHI to achieve OD_600_ = 0.1 (~10^6^ cfu/mL) [31].

For the biofilm inhibition experiments, both unpolarized and polarized PETG/BaTiO_3_NPs (0 wt%, 10 wt%, 20 wt%, and 30 wt%) were utilized. To ensure sterility, both unpolarized and polarized samples underwent sterilization by being immersed in 75% ethanol for one hour and subsequently air-dried under UV light exposure inside a biological safety cabinet. Each sample was then individually submerged in 3 mL of the diluted bacterial solution. To facilitate the adherence of bacteria to the surfaces of the material samples, they were statically incubated at 37 °C for a period of two hours. Subsequently, the samples were transferred to a centrifuge tube containing fresh media and placed in an ultrasonic vibrator. During the incubation period, the samples were subjected to cyclic loading at a frequency of 10 Hz. Take apart the samples after coculture and gently washing them with 1 mL of PBS to remove unattached cells. The biofilm present on the sample surface was then collected using a scraper and placed in fresh BHI medium. The resulting solution was subsequently diluted at three different concentrations, and the number of colonies (unit: CFU) was measured by incubating the samples on BHI agar plates at 37 °C for 24 h. To assess the biomass of the surface biofilms, crystal violet (CV) assays were utilized. To conduct biomass measurements, the biofilm on both unpolarized and polarized samples was treated with 1 mL of a 0.1% CV solution at room temperature for a duration of 20 min. The samples were then subjected to a destaining process involving the addition of 30% acetic acid and shaking at room temperature until the biofilm was completely dissolved. The resulting solution was subsequently transferred to a 96-well plate, and the absorbance at 620 nm was measured in order to quantify the amount of biomass present. The potential antibacterial mechanism of piezoelectric charges was investigated by assessing the accumulation of reactive oxygen species (ROS). To this end, the PETG diaphragm, unpolarized 30 wt% PETG/BaTiO_3_NPs composite diaphragm, and polarized 30 wt% PETG/BaTiO_3_NPs composite diaphragm were co-cultured with bacterial solution for a duration of 30 min at 37 °C. Subsequently, 1 mL of nitrotetrazolium blue chloride (NBT) solution, with a concentration of 1 mg/mL, was added to the bacterial solution. This was performed to evaluate the level of ROS accumulation. At the same time, ROS accumulation was evaluated to assess the potential antibacterial mechanism of piezoelectric charges. After the samples (PETG diaphragm, unpolarized 30 wt% PETG/BaTiO_3_NPs composite diaphragm and polarized 30 wt% PETG/BaTiO_3_NPs composite diaphragm) were cocultured with bacterial solution for 30 min at 37 °C, 1 mL of nitrotetrazolium blue chloride (NBT) solution at a concentration of 1 mg/mL was added to the bacterial solution. To halt the reaction, 0.1 mL of hydrochloric acid was added to the solution. The suspension was then centrifuged for 10 min to obtain bacterial cells after the supernatant was removed. Subsequently, dimethyl sulfoxide (DMSO) was utilized to extract the nitrotetrazolium blue chloride (NBT) present inside the cells, which had been reduced. The NBT was then diluted, and the absorbance at 540 nm under UV light was measured to assess intracellular oxidative pressure.

### 2.6. In Vitro Cytotoxicity

Firstly, we conducted a cell toxicity test using the CCK-8 assay. Unpolarized and polarized PETG/BaTiO_3_NPs (30 wt%) composite diaphragms were selected as the experimental group and PETG diaphragms as the control group. Prior to testing, all samples were immersed in 75% alcohol for 24 h. Following this, both sides of the diaphragm were exposed to UV disinfection for 1 h and subsequently washed with a sterile PBS solution. Extract-based cytotoxicity screening assays were conducted on L-929 cells (L-929 cells: Shanghai Cell Bank, Chinese Academy of Sciences, Shanghai, China) to determine the potential toxicity of the materials. Cells were incubated in DMEM (Shanghai, China) at 37 °C in a humidified atmosphere of 5% CO_2_ until 80–90% confluence. To perform extract testing according to ISO guidelines for surface area/volume (3 cm^2^/mL), both the unpolarized and polarized PETG/BaTiO_3_NPs (30 wt%) composite diaphragm and the control PETG diaphragm were immersed in serum-free DMEM. For cytotoxicity screening, cells were seeded in 100 μL of culture media until adherent (5000 cells/ well in 96-well plates). The next day, the culture media was replaced with 90 μL of fresh media, and 10 μL of extracts from the materials were added for a treatment time of 24 h, 48 h, and 72 h. After treatment, 10 μL of CCK-8 reagent (purchased from Sigma-Aldrich, Yeasen Biotech Co., Ltd., Shanghai, China) was added to 90 μL of fresh serum-free media. The culture plates were then incubated in an incubator at 37 °C and 5% CO_2_ for 2 h, and the absorbance at 450 nm was measured with an enzyme marker. The experiment was conducted independently three times in triplicate.

To further evaluate the cytotoxicity of the composite material, we conducted live/dead staining cytotoxicity experiments. Cells were seeded in a 6-well plate (~5000 cells/well) and cultured in a 37 °C, 5% CO_2_ incubator until they adhered to the wall. The old culture medium was then removed, and 90 μL of fresh DMEM high-glucose culture medium containing fetal bovine serum was added to each well, along with 10 μL of the extracted liquid culture medium. The cells were then cocultured with the materials for 24 h, 48 h, and 72 h. The culture medium was removed from the 6-well plate, and the cells were thoroughly washed three times with prepared PBS buffer to remove any residual esterase activity. Next, 100 μL of Calcein-AM/PI staining working solution was added to the wells, covering the bottom of the plate, and incubated at 37 °C for 15 min. After discarding the staining solution, the cells were washed three times with PBS to remove the fluorescence reagents. The number of live cells (yellow-green) was detected using an inverted fluorescence microscope with a blue excitation light filter, while the number of dead cells was observed using a green excitation light filter. The effect of the materials on the activity of L-929 cells was analyzed.

## 3. Results and Discussion

### 3.1. Characterization

**Figure 2:**
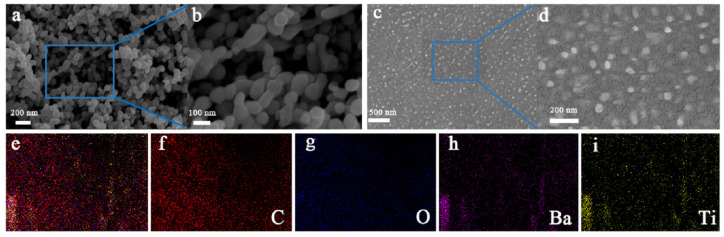
(**a**,**b**) SEM images of BaTiO_3_NPs; (**c**,**d**) SEM images of the cross-section of PETG/BaTiO_3_NPs (30 wt%) composite; (scale (**a**) = 200 nm; (**b**) = 100 nm; (**c**) = 500 nm; (**d**) = 200 nm); (**e**–**i**) element mapping for C, O, Ba, and Ti.

Figure 2 presents the SEM images of BaTiO_3_NPs and PETG/BaTiO_3_NPs composites. In Figure 2a,b, a random distribution of BaTiO_3_NPs was observed, and they are agglomerated. Figure 2c,d present the SEM images of PETG/BaTiO_3_NPs (30 wt%), and uniformly distributed BaTiO_3_NPs in the PETG matrix was observed. Figure S1 shows a scanning electron microscopy (SEM) image of BaTiO_3_ nanoparticles, and the size distribution data of the nanoparticles. It can be seen that the BaTiO_3_ nanoparticles generally have an irregular sphere morphology with an average size of ~100 nm. Moreover, the element mapping of C, O, Ba, and Ti presented by randomly selected positions in Figure 2e directly provides sufficient evidence for the uniform distribution and existence of BaTiO_3_NPs. In Figure 2f–i, the mappings of C, O, Ba, and Ti elements are shown in red, blue, pink, and yellow, respectively.

Figure S2a shows the XRD data of PETG and PETG/BaTiO_3_NPs composites. The most intense peak of the composites was obtained at 31.6°, with miler indices 101, and other miler indices are (001), (101), (111), (002), (102), (112), (202), (003), (103), and (113), respectively [25]. As the strong peak of BaTiO_3_NPs masks the peak of the polymer PETG, the XRD data of the composites do not show the peaks of PETG. The PETG/BaTiO_3_NPs composites and PETG were also characterized by Raman spectroscopy, and the Raman spectra are shown in Figure S2b, with the distinct bands of PETG observed at 477 cm^−1^. No other element-related Raman peaks were observed. However, the distinct bands of PETG/BaTiO_3_NPs composites were observed at 477, 633, 703, and 859 cm^−1^. The addition of BaTiO_3_NPs to the PETG resulted in other peaks. The peaks of PETG/BaTiO_3_NPs composites are usually associated with the asymmetric vibration of the octahedra [32].

### 3.2. Contact Angle and Salivary Absorption

**Figure 3:**
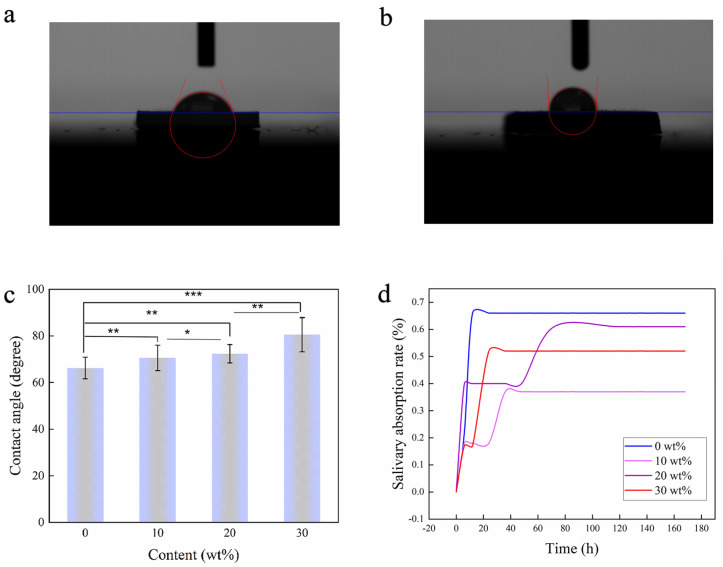
(**a**) Water contact angle image of PETG/BaTiO_3_NPs (0 wt%). (**b**) Water contact angle image of PETG/BaTiO_3_NPs (30 wt%). (**c**) Measurement results of PETG/ BaTiO_3_NPs (0 wt%, 10 wt%, 20 wt%, 30 wt%) water contact angles; (**d**) salivary dissolution rates of PETG/BaTiO_3_NPs (0 wt%, 10 wt%, 20 wt%, 30 wt%). (* *p* < 0.05, ** *p* < 0.01, *** *p* < 0.001).

As shown in Figure 3a–c, the contact angle of the composite surface increases with the increase in BaTiO_3_NPs content, and when the test lasted for 10 min, the contact angles of each group were 66.2 ± 4.7°, 70.6 ± 5.5°, 72.3 ± 3.9°, and 80.5 ± 7.3°. Wettability is one of the most important characteristics of solid surfaces, and the wetting phenomenon is the macroscopic result of microscopic properties of solid surface properties, liquid properties, and molecular interactions between solid-liquid interfaces [33]. Additionally, according to the existing studies, bacterial adhesion is firmly related to the surface wettability [34]. Prabhu Suraj Nanduru et al. [35] found that the hydrophobic surface can inhibit biofilm formation. The antibacterial results in this study suggest that a relatively hydrophobic surface is more effective at inhibiting the adhesion and reproduction of bacteria.

During the process of infiltrating the invisible appliance in the oral saliva environment, saliva absorption is likely to occur, affecting the performance of the invisible appliance. Figure 3d shows the curve of the saliva absorption rate of PETG/BaTiO_3_NPs (0 wt%, 10 wt%, 20 wt%, and 30 wt%) over time. The results showed that both PETG and the PETG/ BaTiO_3_NPs composites had a small amount of salivary absorption during the initial immersion in saliva. However, the saliva absorption rate of the composite was lower than that of the PETG, and all sample materials reached a stable state after approximately 3 days of immersion. The final salivary absorption rates of PETG/ BaTiO_3_NPs (0 wt%, 10 wt%, 20 wt%, and 30 wt%) at 7 days were 0.66%, 0.37%, 0.61%, and 0.52%, respectively.

### 3.3. Mechanical Properties

Statistical results in Figure S4a show that the microhardness of PETG/BaTiO_3_NPs (10 wt%, 20 wt%, and 30 wt%) composites was higher than PETG diaphragm, but the difference was not statistically significant, and the increase in microhardness was not concentration dependent. The results of Figure S4b show that the yield strength and tensile strength of PETG/BaTiO_3_NPs (10 wt%, 20 wt%, and 30 wt%) composites are higher than those of PETG diaphragm.

### 3.4. In Vitro Antibacterial Test

**Figure 4:**
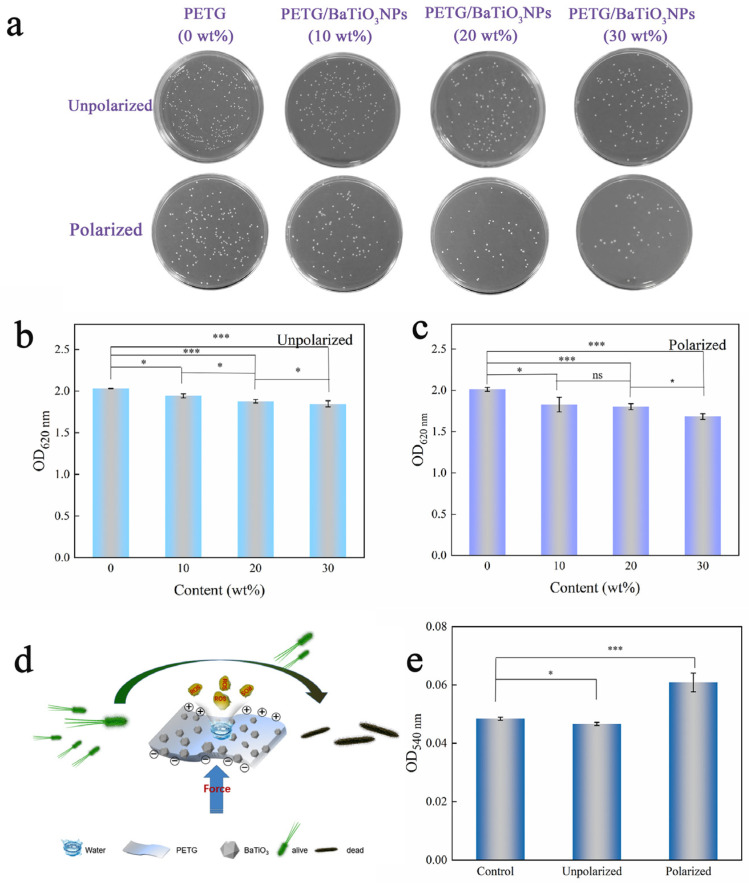
(**a**) Photographs of *Streptococcus mutans* colonies on the agar plate of unpolarized and polarized PETG/ BaTiO_3_NPs (0 wt%, 10 wt%, 20 wt%, 30 wt%); (**b**,**c**) Quantitative results of surface biofilm of the unpolarized and polarized PETG/BaTiO_3_NPs (0 wt%, 10 wt%, 20 wt%, 30 wt%); (**d**) Schematic of the antibacterial principle of polarized composites; (**e**) Quantitative results of intracellular ROS concentration. (* *p* < 0.05, *** *p* < 0.001, ns = no significance).

Figure 4d shows the antibacterial principle of a polarized composite diaphragm. The surface of nanocomposite materials generates an electric charge under micromechanical stimulation, and the electric charge interacts with bacteria to inhibit their growth and adhesion. Figure 4a shows the count results of the antibacterial colony plate of unpolarized and polarized PETG/BaTiO_3_NPs composites (0 wt%, 10 wt%, 20 wt%, and 30 wt%). The results show that the unpolarized composite diaphragm has a certain antibacterial effect, and with the increasing BaTiO_3_NPs content, the antibacterial effect was gradually enhanced, and the antibacterial rate of the composites containing 10 wt%, 20 wt%, and 30 wt% BaTiO_3_NPs could reach 19.35 ± 4.35%, 37.64 ± 5.75%, and 42.90 ± 3.72%, respectively. The results also show that the count results of the bacterial colony plates of polarized PETG/BaTiO_3_NPs (0 wt%, 10 wt%, 20 wt%, and 30 wt%) increased the BaTiO_3_NPs content, the antibacterial effect was gradually enhanced. The antibacterial rate of the polarizing composites containing 10 wt%, 20 wt%, and 30 wt% BaTiO_3_NPs reached 41.65 ± 2.34%, 63.15 ± 4.98%, and 67.39 ± 5.35%, respectively (Figure S3). The polarized PETG/BaTiO_3_NPs composites showed a better antibacterial effect than the unpolarized ones. In order to further verify this antibacterial result, Figure 4b,c show the quantitative results of the surface biomass of unpolarized and polarized PETG/BaTiO_3_NPs diaphragm (0 wt%, 10 wt%, 20 wt%, and 30 wt%), which are consistent with the results of colony plate counting, indicating that unpolarized BaTiO_3_NPs has certain antibacterial properties. The polarized PETG/BaTiO_3_NPs composites showed a better antibacterial effect than the unpolarized ones. Aadil Abass Shah et al. [26] also found that BaTiO_3_NPs had good antibacterial activity and antibacterial film performance. In a word, the results showed that the polarized PETG/BaTiO_3_NPs had good bacteriostatic performance under micromechanical stimulation. In order to further explore the mechanism of this antibacterial result, Figure 4e shows ROS concentrations of PETG (control), unpolarized PETG/BaTiO_3_NPs (30 wt%) (unpolarized), and polarized PETG/BaTiO_3_NPs (30 wt%) (polarized) composites after coculture with bacteria under mechanical stimulation. This result indicates that when the PETG/BaTiO_3_NPs complex is subjected to mechanical stimulation after polarization, ROS is produced in *S. mutans* cells, indicating that mechanical stimulation could activate the charge on the surface of the polarized PETG/BaTiO_3_NPs composites. Previous studies have found that piezoelectric materials have a unique piezoelectric effect. When subjected to external mechanical stress, piezoelectric materials can immediately generate an internal electric field and surface voltage potential [16]. Moreover, studies have found that piezoelectric materials are very sensitive to mechanical vibration, and water flow, muscle movement, and breathing can induce the generation of surface charge [28,29,30]. Therefore, when subjected to slight mechanical stimulation caused by daily oral activities (such as drinking, talking, exercising, etc.) in the mouth, the material surface can be induced to generate electric charges, and the surface molecules of bacterial cells are electrolyzed to produce reactive oxygen species (ROS). Meanwhile, these surface charges also contribute to the production of intracellular ROS. Such as superoxide anion (O^2−^), hydrogen peroxide (H_2_O_2_), hydroxyl radical (•OH), lipid hydroperoxide, and singlet oxygen (O^2^_1_) [33]. The increase in reactive oxygen species exceeds the antioxidant capacity of cells, resulting in oxidative stress, destruction of lipids, proteins, and DNA, and ultimately the death of bacterial cells. The experimental results of this work also indicate that the polarized composites may activate surface charges and produce ROS under micromechanical stimulation, showing excellent antibacterial effects.

### 3.5. Cytotoxicity toward L-929

**Figure 5:**
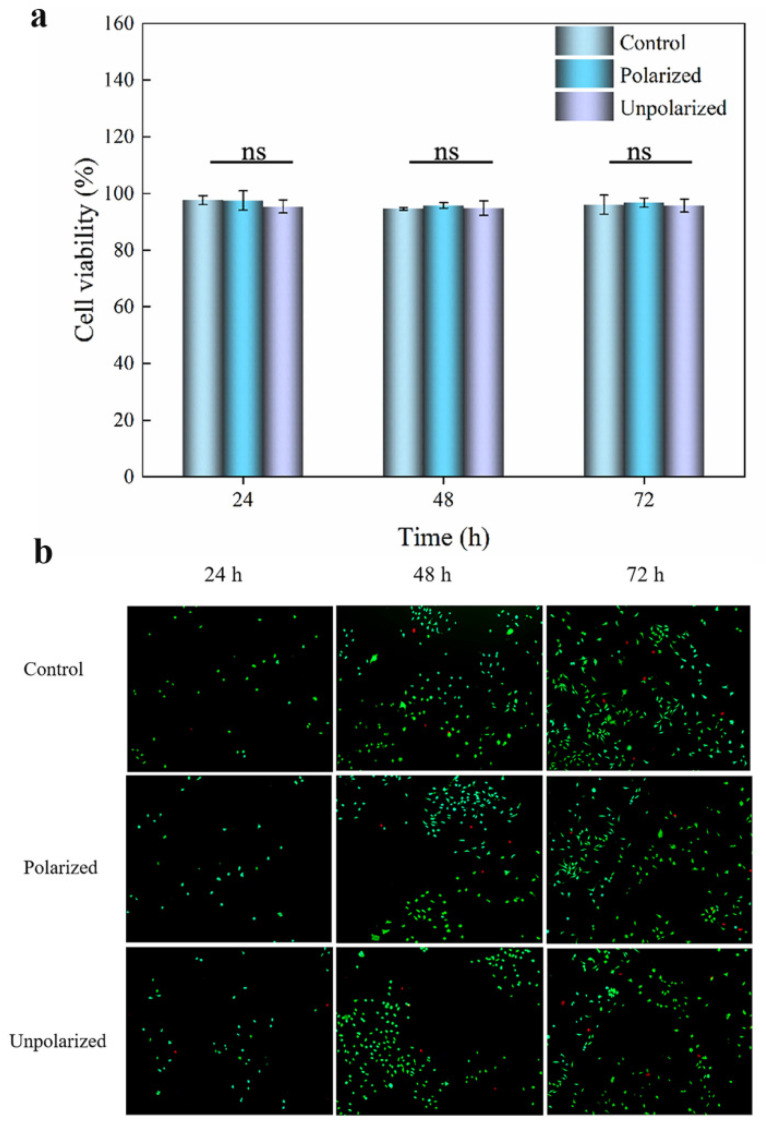
Cytotoxicity of PETG (control), polarized PETG/BaTiO_3_NPs (30 wt%), and unpolarized PETG/BaTiO_3_NPs (30 wt%) toward L-929 cells. (**a**) Percentage cell viability for extract test-based cytotoxic screening of control, polarized, and unpolarized groups. (**b**) Live-dead staining images of L-929 cells co-cultured with extraction solutions from PETG, polarized and unpolarized PETG/BaTiO_3_NPs (30 wt%) diagram at 24, 48, and 72 h (scale = 200 μm). (ns = no significance).

We studied the antibacterial activity of PETG/BaTiO_3_NPs composite discs against an oral pathogen (*S. mutans*), and cytotoxicity testing is essential. In this work, L-929 cells were selected for the cytotoxicity of PETG/BaTiO_3_NPs (30 wt%) composite discs. Here, we followed ISO guidelines to perform an extract cytotoxicity test on PETG/BaTiO_3_NPs (30 wt%) composite discs and control discs. The effect of the PETG/BaTiO_3_NPs composite discs on the viability of L-929 cells was evaluated using the CCK-8 assay. In Figure 5a, the data from CCK-8 assays showed that the viabilities of L-929 cells cocultured with the extract media (media in contact with samples for 24 h at 37 °C and 5% CO_2_) demonstrated no significant drop in cell viability for the cell line with treatment times of 24 h, 48 h, and 72 h. After 72 h of coculture, the cells in the control, unpolarized, and polarized groups maintained a high viability of 96.02 ± 3.38%, 96.74 ± 1.6%, and 95.67 ± 2.3%, respectively. In Figure 5b, the results of the live-dead staining cell toxicity experiment showed that after 24 h of exposure to each sample in the extraction solution culture medium (cultured at 37 °C and 5% CO_2_), a large number of live cells (represented by green fluorescence) and a small number of dead cells (represented by red fluorescence) were visible in the fields of view of the PETG (control), polarized PETG/BaTiO_3_NPs (30 wt%, polarized), and unpolarized PETG/BaTiO_3_NPs (30 wt%, unpolarized) groups. However, there was no statistically significant difference in the number of live and dead cells among the three groups. After 48 h of treatment with the extraction solution culture medium, a large number of live cells and a small number of dead cells were visible in the fields of view of the PETG (control), polarized PETG/BaTiO_3_NPs (30 wt%, polarized), and unpolarized PETG/BaTiO_3_NPs (30 wt%, unpolarized) groups, and the number of live and dead cells increased significantly compared to 24 h, but there was still no statistically significant difference among the three groups. After 72 h of treatment with the extraction solution culture medium, a large number of live cells and a small number of dead cells were visible in the fields of view of the PETG (control), polarized PETG/BaTiO_3_NPs (30 wt%, polarized), and unpolarized PETG/BaTiO_3_NPs (30 wt%, unpolarized) groups, and there was no statistically significant difference in the number of live and dead cells among the three groups. Furthermore, previous research has also shown that BaTiO_3_NPs can serve as a platform for biomaterials [27,32]. Similarly, a new adhesive reported by Gang Ge et al. has high biocompatibility, good sealing ability, and excellent adhesion [36]. These materials with good biocompatibility have a good application prospect in bioengineering and medical implants.

## 4. Conclusions

In this study, we have introduced piezoelectric nanoparticles (BaTiO_3_) into PETG and evaluated their potential to inhibit pathogenic biofilm growth at the interface between teeth and orthodontic aligners. Our findings suggest that these composites have significant antibacterial properties, resulting in a marked decrease in biofilm biomass and viable bacteria when compared to the control. In the meantime, we also discovered that the antimicrobial activity of the composites can be affected by changes in filler content and the polarization of the piezoelectric composite. This work demonstrates the potential of piezoelectric BaTiO_3_NPs as a novel material for combating pathogenic bacteria in thermoplastic orthodontic appliances. The composite materials offer several advantages, such as long-term delivery of therapeutic effects, no concern over the increased bacterial resistance to drugs, no leaching of the compounds, and biocompatibility. This study provides new ideas for the development of invisible antibacterial appliances.

## Data Availability

The experimental data presented in the present paper are available from the corresponding author upon request.

## Supplementary Materials

The following supporting information can be downloaded at: https://www.mdpi.com/article/10.3390/s23115336/s1, Figure S1: SEM images of BaTiO_3_NPs and and the inset shows the particle size distribution of BaTiO_3_NPs; Figure S2: (a) X-ray diffraction images of PETG and PETG/BaTiO_3_NPs (30 wt%) composites; (b) Raman spectra of PETG and PETG/BaTiO_3_NPs (30 wt%) composites.; Figure S3: (a) Antibacterial rates against *S. mutans* of the unpolarized PETG/BaTiO_3_NPs (0 wt%, 10 wt%, 20 wt%, 30 wt%); (b) Antibacterial rates against *S. mutans* of the polarized PETG/BaTiO_3_NPs (0 wt%, 10 wt%, 20 wt%, 30 wt%); Figure S4: (a) microhardness of PETG/BaTiO_3_NPs(0 wt%, 10 wt%, 20 wt%, 30wt%); (b) Stress-strain curves of PETG/BaTiO_3_NPs (0 wt%, 10 wt%, 20 wt%, 30wt%).
