# Supplementary material for: Preparation of Nanocomposites for Antibacterial Orthodontic Invisible Appliance Based on Piezoelectric Catalysis
Source: Sensors (Basel). 2023 Jun 5;23(11):5336. doi: 10.3390/s23115336 (PMC10256112; doi:10.3390/s23115336)
Supplement: Supplementary file 1 [file sensors-23-05336-s001.zip › sensors-2382319-supplementary.pdf]

Supporting Information

Preparation of Nanocomposites for Antibacterial Orthodontic Invisible Appliance Based on Piezoelectric Catalysis

S1. Particle size distribution

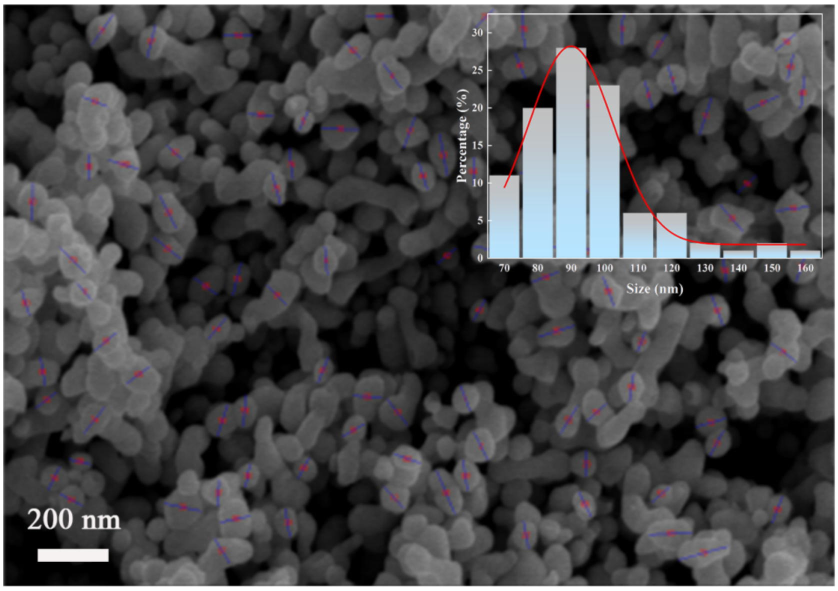

Figure S1 SEM images of $BaTiO_3$NPs and and the inset shows the particle size distribution of $BaTiO_3$NPs

S2. X-ray diffraction and Raman spectra

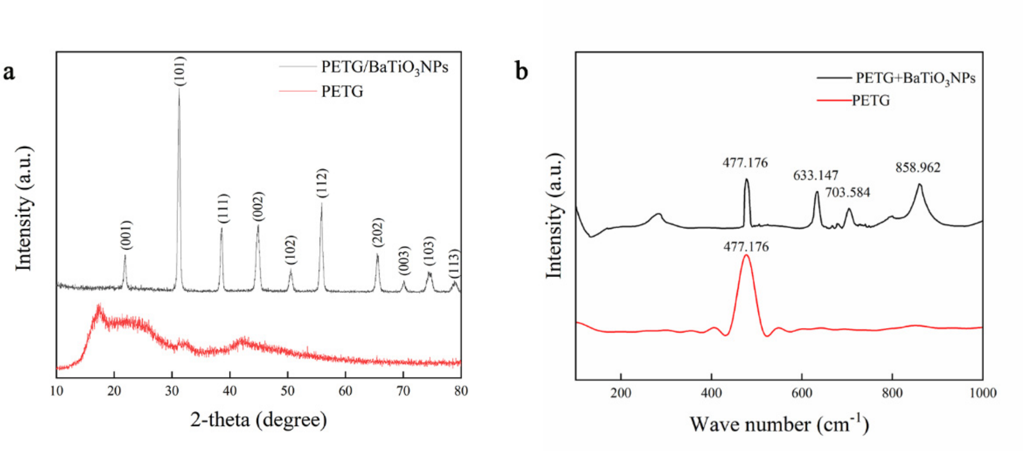

Figure S2 (a) X-ray diffraction images of PETG and PETG/$BaTiO_3$NPs (30 wt%) composites; (b) Raman spectra of PETG and PETG/$BaTiO_3$NPs (30 wt%) composites.

S3. Antibacterial test

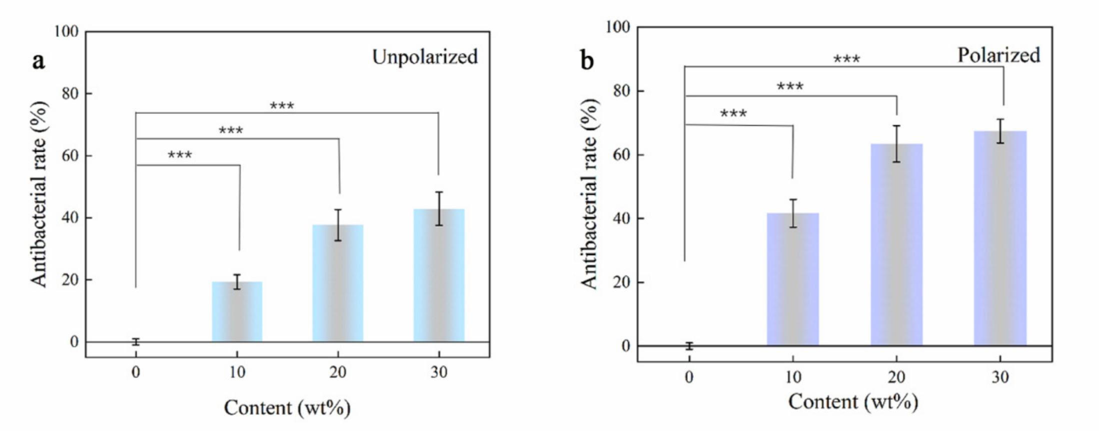

Figure S3 (a) Antibacterial rates against S. mutans of the unpolarized PETG/$BaTiO_3$NPs (0 wt%, 10 wt%, 20 wt%, 30 wt%); (b) Antibacterial rates against S. mutans of the polarized PETG/$BaTiO_3$NPs (0 wt%, 10 wt%, 20 wt%, 30 wt%) (****P*<0.001)

S4. Mechanical properties

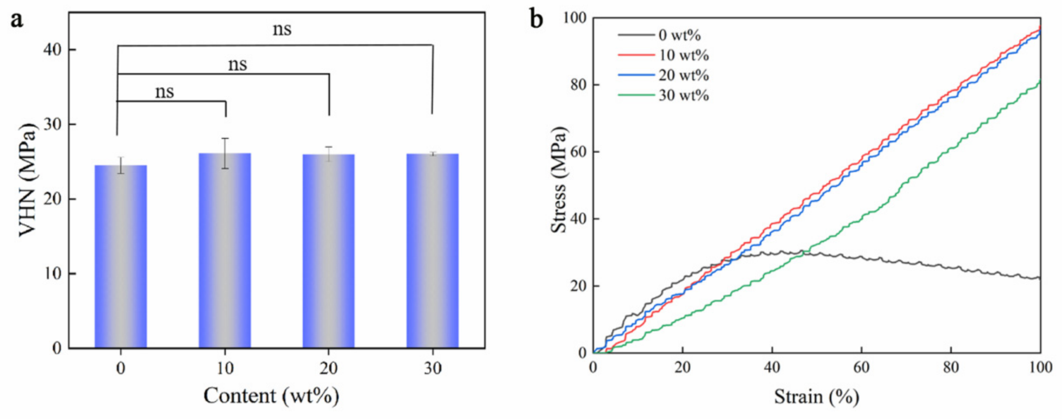

Figure S4 (a) microhardness of PETG/$BaTiO_3$NPs(0 wt%, 10 wt%, 20 wt%, 30wt%); (b) Stress-strain curves of PETG/$BaTiO_3$NPs(0 wt%, 10 wt%, 20 wt%, 30wt%) (ns=no significance)
